# Novel applications of gene and cell therapies in the treatment of gynecological disorders

**DOI:** 10.3389/fmed.2025.1658357

**Published:** 2025-10-08

**Authors:** Huiping He, Chufan Zhou, Jian Xiong, Jiaying Fan

**Affiliations:** Department of Obstetrics and Gynecology, Guangzhou Women and Children’s Medical Center, Guangzhou Medical University, Guangzhou, China

**Keywords:** natural killer cells, gene modulation, gynecological disorders, human papillomavirus, endometriosis, mesenchymal stem cells, gene and cell therapies

## Abstract

Gynecologic disorders, such as cervical and ovarian tumors, uterine fibroids, and endometriosis, present significant clinical challenges due to frequent recurrence, emerging chemoresistance, and undesirable effects associated with prolonged hormonal treatments. Conventional therapies often fail over time as tumors develop resistance through mechanisms that include the inactivation of tumor suppressor genes, immune evasion, and activation of oncogenic signaling pathways. These limitations underscore the urgent need for more precise and durable therapeutic strategies. Gene- and cell-based therapies have emerged as promising next-generation approaches to address these challenges. CRISPR/Cas9-based editing, RNA-directed regulation, and targeted gene modulation are being employed to silence resistance genes, restore tumor suppressors, and resensitize tumors to platinum (Pt)-based chemotherapy, hormonal therapy, and immunotherapy. Delivery platforms such as receptor-targeted lipid nanoparticles (LNPs) and viral vectors (VVs) enhance tissue specificity and improve therapeutic efficacy. Concurrently, advanced immune cell therapies, including modified natural killer (NK) cells and CAR-T cells, are being designed to eradicate tumor clones that evade standard therapeutic approaches. For benign gynecologic conditions, mesenchymal stem cells (MSCs) demonstrate regenerative potential and may offer an alternative to repeated surgical interventions or prolonged hormonal suppression. This review summarizes recent trends in gene and cell therapies for uterine disorders and gynecologic cancers, with a focus on their potential to enhance therapeutic efficacy, overcome drug resistance, and preserve reproductive health.

## 1 Introduction

Malignant and benign gynecological disorders have a significant impact on reproductive health and overall quality of life. Cervical cancer is the fourth most common cancer among women. There were approximately 570,000 new cases and 311,000 deaths worldwide. The burden is particularly heavy in low- and middle-income countries (LMICs) with limited screening and vaccination resources (1). Despite the availability of preventive HPV vaccines and effective screening methods (such as Pap smears and HPV testing), the mortality rate remains high due to delayed diagnosis and the difficulty in accessing radical treatments such as surgery and radiotherapy in resource-poor areas (2, 3). The main cause is persistent infection with high-risk human papillomavirus (HPV), especially HPV16 and HPV18 types, which drive cell carcinogenesis through E6 and E7 oncogenic proteins (4).

In contrast, although ovarian cancer has a relatively low incidence rate (about 314,000 cases per year), it is the gynecological malignancy with the highest fatality rate. The main reason is that 70% of the cases are already at stage III or IV at the time of diagnosis, and the recurrence rate is high (5–7). Standard treatment includes cytoreductive surgery combined with platinum-based chemotherapy; However, up to 70% of patients in the advanced stage will experience a recurrence of platinum resistance, resulting in a low long-term survival rate (the 5-year survival rate for stage III/IV patients is less than 30%) (8). This drug resistance is usually mediated by multiple mechanisms, such as enhanced drug excretion through ABC transporters and immune escape induced by the tumor microenvironment (9).

The impact of benign gynecological diseases is equally significant. Endometriosis affects approximately 10% of women of childbearing age, causing chronic pelvic pain, dysmenorrhea and infertility (10). First-line treatment includes hormone therapy (such as compound oral contraceptives, progesterone, GnRH agonists), but it is often limited due to obvious side effects and a high recurrence rate after drug withdrawal (up to 50% within 5 years) (11). Similarly, the incidence of uterine fibroids (leiomyomas) among women under the age of 50 is as high as 70%, which can cause menorrhagia, pelvic pressure and reproductive dysfunction (12). Although surgical treatments (myomectomy, hysterectomy) are effective, they carry the risk of complications and cannot prevent recurrence, especially among young women (13).

Overall, these diseases highlight several key unmet clinical needs: (1) The need for more effective early detection strategies for ovarian cancer; (2) Overcome the problem of drug resistance in the treatment of cervical cancer and ovarian cancer; (3) Develop non-surgical and persistent treatment methods for hormone-resistant endometriosis and recurrent fibroids; (4) Achieve fair accessibility of preventive and therapeutic interventions in different medical resource contexts. Gene and cell therapy offers a highly promising approach to addressing these challenges, enabling precise intervention at the molecular level—for instance, silencing drug-resistant genes (such as BCL2, MDR1), restoring the function of tumor suppressor genes (such as TP53), or regulating inflammatory pathways involved in the progression of endometriosis. For instance, preclinical studies have shown that knocking out BCL2 through CRISPR/Cas9 technology can enhance the sensitivity of ovarian cancer cells to chemotherapy (14), while gene editing of inflammatory mediators may reduce the activity of endometriosis lesions (15).

This work highlights the incorporation of gene and cell therapies to treat gynecological disorders, focusing on their potential to control drug resistance, improve therapeutic accuracy, and support reproductive function.

## 2 Gene therapy in gynecological disorders

Gene therapy has become a transformative approach in modern medicine, capable of targeting and correcting or regulating pathogenic genes in various diseases such as single-gene disorders, cancers, and chronic inflammatory diseases (16). By introducing, silencing or editing specific gene sequences, gene therapy can directly intervene in the molecular root cause of diseases, rather than merely alleviating symptoms. Key technologies such as CRISPR/Cas9 genome editing, RNA interference (RNAi), and gene replacement have demonstrated clinical success in hematological malignancies, hereditary retinal diseases, and neuromuscular diseases (17). These advancements highlight the high flexibility and precision of gene therapy in diseases driven by clear genetic alterations or dysregulation of signaling pathways.

In the context of gynecological diseases (including benign and malignant ones), gene therapy offers a highly promising strategy to address long-term challenges such as treatment resistance, recurrence, and short-lived efficacy. Its ability to precisely target carcinogenic drivers, restore the function of tumor suppressor genes and regulate the inflammatory microenvironment makes it possible for it to become a paradigm shift in the treatment models of cervical cancer, ovarian cancer, endometriosis and uterine fibroids. The following sections will outline the main gene therapy strategies currently being explored in these diseases, with a focus on the CRISPR/Cas9 system, delivery platforms, and molecular targets.

### 2.1 CRISPR/Cas9-based therapeutics

The CRISPR/Cas9 system is a programmable genome editing tool that uses guide RNA (gRNA) to direct Cas9 nuclease to target specific DNA sequences and induce double-strand breaks (DSBS) at this point. This break can be repaired through non-homologous end joining (NHEJ) or homologous directed repair (HDR) mechanisms, thereby achieving gene knockout, correction or insertion.

The CRISPR/Cas9 system enables the precise targeting and removal of resistance pathways in gynecological diseases that disrupt the E6 and E7 oncogenes, promoting apoptosis, restoring p53 and Rb function, and enhancing sensitivity to radiotherapy and cisplatin. In ovarian cancer, drug resistance mechanisms involve ABCB1 overexpression and BRCA1/2 dysfunction, both of which challenge genomic repair precision. The efflux of doxorubicin and paclitaxel is reduced by eliminating ABCB1 through CRISPR/Cas9 editing, thereby improving their therapeutic potency (18, 19).

Moreover, repairing BRCA1 or TP53 mutations via CRISPR in HR-deficient malignancies re-establishes sensitivity to Pt compounds and PARP inhibitors. These studies highlight the dual role of gene editing in both suppressing disease progression and restoring chemotherapeutic efficacy. Innovative strategies that integrate RISPR with pharmacological agents are being adopted to enhance the effectiveness of killing tumor cells (20).

### 2.2 Gene delivery platforms: viral and non-viral vectors

Gene delivery is a key bottleneck in gene therapy, which requires vectors that can efficiently transport genetic material to target cells while minimizing immune activation and off-target effects. Vectors are mainly classified into viral vectors (such as AAV and lentiviruses) and non-viral vectors (such as lipid nanoparticles and polymers), each having different advantages in terms of transduction efficiency, payload capacity and safety.

Effective gene therapy relies strongly on the development of benign and efficient delivery vehicles. Viral vectors, particularly adeno-associated viruses (AAVs), remain the most validated platforms, offering sustained expression of therapeutic genes in both malignant and benign reproductive tissues. AAV-mediated delivery of shRNAs targeting HPV-E7 has resulted in durable tumor regression and enhanced chemo-radio-sensitivity in cervical cancer models (21).

In endometriosis, AAVs are employed to deliver anti-inflammatory cytokines, such as IL-10, which modulates local immune responses and potentially enhances sensitivity to hormonal treatments. Targeted gene delivery also holds potential in fibroid management, where gene silencing of TGF-β receptors may improve responsiveness to selective progesterone receptor modulators (SPRMs) (22).

Non-viral delivery systems provide greater customization for therapeutic applications and exhibit lower immunogenic potential compared to viral vectors (23). These vectors are functionalized with targeting ligands to enable selective delivery of therapeutic payloads to endometrial and ovarian tissues. The development of dual-function nanoparticles has shown the delivery of siRNAs targeting resistance pathways and chemotherapeutics, as exhibited by the co-delivery of paclitaxel and BCL2 siRNA to treat multidrug-resistant ovarian cancer (24).

### 2.3 Targeting driver oncogenes and tumor suppressor genes

Oncogenes and tumor suppressor genes are the two basic categories of cancer drivers. Oncogenes (such as PI3K and AKT) acquire functions through mutations or overexpression, promoting cell survival and proliferation. However, tumor suppressor genes (such as TP53 and BRCA1) lose their function, leading to uncontrolled cell growth. Targeting these genes through gene silencing or alternative strategies provides a reasonable path to correct the molecular imbalance behind gynecological tumors and benign proliferative diseases.

Gynecologic cancers exhibit aberrant activation of oncogenic drivers and frequently harbor inactivating mutations in tumor suppressor genes, which contribute to disease growth and therapeutic resistance. Gene therapy can reinstate therapeutic responsiveness and potentiate the effects of co-administered treatments by directly targeting abnormal genetic pathways (25).

Therapeutic restoration of wild-type p53 has improved the responsiveness to radiotherapy and cisplatin in both cervical and endometrial cancers (26). Similarly, silencing of PI3K/Akt/mTOR via siRNA or shRNA has enhanced doxorubicin-induced apoptosis in ovarian cancer models and reversed pro-survival signaling. In hormone-resistant endometriosis, suppression of SF-1, COX-2, and IL-6 via RNA interference (RNAi) has been shown to reduce lesion volume and improve sensitivity to progesterone therapy (27).

## 3 Cell therapy in gynecological disorders

Cell-based therapies are gaining attention in gynecologic medicine, not only as standalone treatments but also as adjuncts to conventional modalities for overcoming drug resistance and immunosuppression. These therapies offer precise immune activation or tissue regeneration, addressing both malignant and non-malignant disorders that often prove refractory to chemotherapy, hormone therapy, or surgery. From engineered immune cells that target chemoresistant tumor clones to regenerative stem cells that restore endometrial integrity, cell therapy opens new avenues in precision medicine (28).

### 3.1 CAR-T and TCR-engineered T cells

CAR-T cells and T cell receptor (TCR)-modified T cells are at the forefront of immunotherapy for solid tumors. In gynecologic cancers, particularly ovarian and cervical, drug resistance frequently arises from antigenic drift, immune escape, or a suppressive tumor microenvironment (TME), which limits the effectiveness of Pt-based drugs, taxanes, and immune checkpoint inhibitors (29).

CAR-T therapies are designed to bypass these resistance mechanisms by recognizing surface antigens independent of MHC presentation. In ovarian cancer, CAR-T cells targeting MUC16 (CA125), mesothelin, and folate receptor-*α* have demonstrated potent activity in preclinical models and early-phase clinical trials (30). Importantly, these therapies may eradicate tumor clones resistant to cisplatin or bevacizumab, thus complementing standard regimens. Dual-targeting CARs, also known as “logic-gated” CARs (e.g., SynNotch systems), are being tested to mitigate off-target toxicity and address tumor heterogeneity.

In HPV-associated cervical cancer, TCR-T cells that recognize viral antigens (e.g., HPV E6/E7) offer a potential route to eliminate tumor cells that are unresponsive to radiotherapy and chemotherapy. These antigen-specific T cells can target intracellular proteins processed on MHC class I, thereby extending the therapeutic reach beyond surface markers and providing a strategy against tumors with defective DNA damage responses or altered p53 (31).

### 3.2 NK cell therapy

NK cells are emerging as effective agents targeting drug-resistant gynecologic tumors, providing innate cytotoxicity prior to sensitization. These cells retain their cytotoxic function against tumor cells that downregulate MHC expression, a common mechanism of immune escape in chemoresistant tumors. Unlike T cells, they are not restricted by MHC molecules (32).

CAR-modified NK cells have been designed to specifically target ovarian cancer-associated antigens, enhancing their targeted cytotoxicity against tumor cells. The use of CAR-NK cells in patients with Pt-resistant ovarian cancers has shown promising results. These include tumor clearance and reduced cytokine-related toxicity, resulting in their safe and effective role. For patients who have relapsed and not responded to drugs such as paclitaxel or bevacizumab, these CAR-NK cell platforms are beneficial, as tumor antigen profiles remain stable, allowing for sustained targeting of the disease (33). Furthermore, NK cells can exert synergistic effects; for instance, beneficial efficacy in endometrial and HER2-positive uterine tumors can be improved by integrating NK cells with anti-HER2 or anti-PD-L1 antibodies.

### 3.3 Mesenchymal stem cells

Due to their immunomodulatory, regenerative, and anti-fibrotic characteristics, MSCs present promising alternatives for treating benign uterine disorders where conventional therapies are ineffective. Circumstances such as uterine synechiae, Asherman’s syndrome, and thin endometrium often arise from the repeated intrauterine procedures or unresponsive endometritis. These cells have demonstrated the potential to restore endometrial thickness and promote angiogenesis through paracrine signaling and direct tissue integration, making them a valuable tool for uterine regeneration (34). This regenerative effect may reverse endometrial damage caused by the prolonged use of GnRH analogues, presenting a fertility-preserving therapeutic alternative for the patients.

Beyond structural repair, MSCs are being used as carriers of therapeutic genes or microRNAs. Engineered MSCs expressing IL-10 or VEGF can attenuate fibrosis and improve hormonal responsiveness in endometriosis. Additionally, exosome-derived MSC therapies are emerging as cell-free options for delivering regulatory RNAs that target inflammation, estrogen metabolism, or fibrotic remodeling, thereby enhancing responsiveness to progesterone therapy or aromatase inhibitors (35).

**Figure 1:**
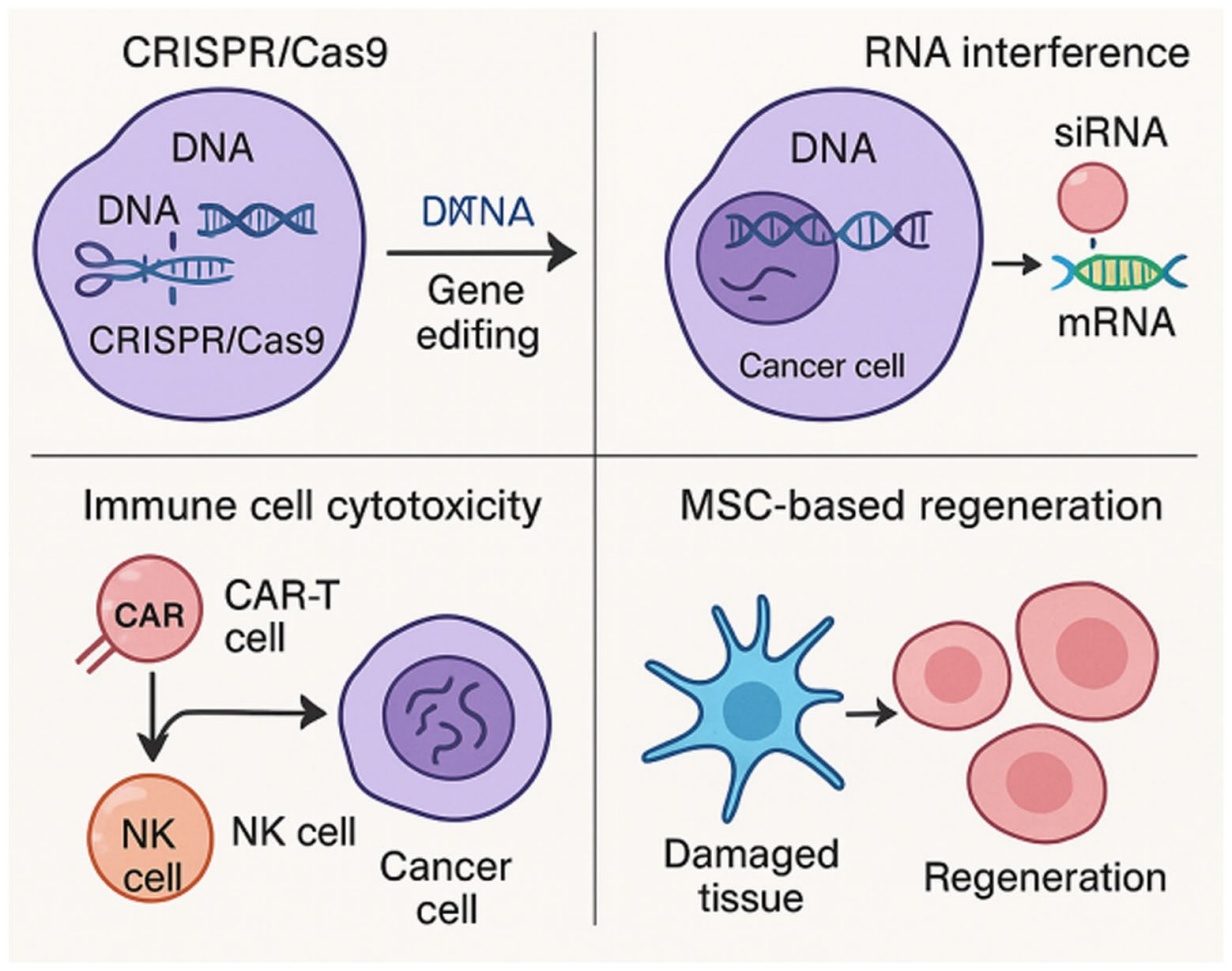
Schematic illustration of key therapeutic mechanisms underlying gene and cell therapies in gynecological disorders, including CRISPR/Cas9-mediated gene editing, RNA interference, immune cell cytotoxicity, and MSC-based regeneration.

Safety concerns related to tumorigenicity, immune rejection, or ectopic differentiation are being closely monitored; however, early-phase clinical trials have demonstrated good tolerability. Scaffold-based delivery systems, autologous sourcing, and controlled gene loading are being developed to improve localization and therapeutic outcomes. Figure 1 presents the main therapeutic mechanisms underlying gene and cell therapies in gynecological disorders, including CRISPR/Cas9-enabled gene editing, RNA interference, immune cell cytotoxicity, and MSC-based regeneration (see Table 1).

**Table 1:** Comparative analysis of gene and cell therapy strategies in gynecological disorders: advantages, challenges, and representative examples.

Strategy	Advantages	Disadvantages/challenges	Examples
CRISPR/Cas9-mediated genome editing	Highly specific targeting of pathogenic genes (e.g., E6/E7, BRCA1); enables gene knockout, repair, or insertion; restores chemotherapy sensitivity.	Off-target effects risk; low HDR repair efficiency; challenges in *in vivo* delivery; may trigger p53-mediated toxicity or immune responses.	Knockout of ABCB1 decreases doxorubicin and paclitaxel efflux in multidrug-resistant ovarian cancer (8, 9); repair of BRCA1 mutations restores sensitivity to platinum-based agents and PARP inhibitors (10).
Viral vectors (e.g., AAV)	High transduction efficiency; enables long-term gene expression in target tissues; clinically validated favorable safety profile.	High immunogenicity (pre-existing antibodies limit repeated administration); limited payload capacity (<4.7 kb); potential risk of insertional mutagenesis.	AAV-delivered shRNA targeting HPV-E7 induces sustained tumor regression in cervical cancer models (11); AAV-mediated IL-10 expression modulates inflammation in endometriosis (12).
Non-viral delivery systems (e.g., nanoparticles)	Scalable production; low immunogenicity; facile functionalization; supports co-delivery (genes + drugs).	Low transduction efficiency; poor *in vivo* stability; susceptible to clearance by the reticuloendothelial system (RES); targeting specificity requires further optimization.	Nanoparticle-mediated co-delivery of BCL2 siRNA and paclitaxel reverses multidrug resistance in ovarian cancer (14); TGF-β receptor siRNA delivery enhances uterine fibroid response to SPRMs (12).
Targeting oncogenes/tumor suppressor genes (via RNAi or gene replacement)	Directly intervenes in core disease driver pathways (e.g., p53 inactivation, PI3K/Akt activation); synergizes with conventional therapies; applicable to diverse gynecological malignancies and benign conditions.	RNAi exhibits off-target and transient effects; gene replacement requires sustained expression; in vivo delivery remains a bottleneck.	Restoration of wild-type p53 enhances sensitivity to radiotherapy and cisplatin in cervical cancer (16); RNAi-mediated silencing of SF-1, COX-2, and IL-6 reduces hormone-resistant endometriotic lesions (17).
CAR-T and TCR-engineered T cells	Specifcally recognizes tumor antigens (MHC-independent); eliminates chemotherapy-resistant clones; dual-targeting CARs address heterogeneity.	Poor solid tumor infiltration; risk of cytokine release syndrome (CRS); on-target/off-tumor toxicity; high manufacturing costs and prolonged production cycles.	CAR-T cells targeting MUC16, mesothelin, and folate receptor-α demonstrate potent antitumor activity in ovarian cancer models (20); TCR-T cells recognizing HPV E6/E7 antigens are utilized for chemoradiation-resistant cervical cancer (21).
NK cell therapy (including CAR-NK)	Innate immune cells, MHC-independent recognition; effective against tumors with low MHC expression; low cytokine toxicity; favorable safety profile.	Poor in vivo persistence; difficult ex vivo expansion; limited homing efficiency to solid tumors; requires exogenous IL-15 support.	CAR-NK cells have achieved tumor clearance with low toxicity in platinum-resistant ovarian cancer patients (23); combination with anti-HER2 or anti-PD-L1 antibodies enhances efficacy in endometrial cancer and HER2 + uterine tumors (23).
Mesenchymal stem cells (MSCs)	Possesses immunomodulatory, tissue regenerative, and antifibrotic properties; applicable in uterine repair (e.g., Asherman syndrome); can serve as gene/exosome delivery vehicles.	Risks of ectopic differentiation, pulmonary sequestration, or tumor-promoting effects; long-term safety not yet fully validated; lack of standardized preparation protocols.	MSCs promote endometrial regeneration, improving thin endometrium and GnRH agonist-induced damage (24); engineered MSCs expressing IL-10/VEGF ameliorate fibrosis and enhance hormonal response in endometriosis (25).

## 4 Emerging technologies and translational challenges

While gene and cell therapies are redefining treatment, several technological and biological challenges limit the clinical translation of paradigms in gynecological medicines. Many of these challenges intersect directly with the issues of drug application and treatment resistance, underscoring the importance of innovative platforms and strategic integration into existing therapeutic regimens.

### 4.1 Synthetic biology and competent vectors

Synthetic biology is enabling the creation of “intelligent” therapeutic platforms, such as logic-gated CAR-T cells, inducible promoters, and tumor-selective gene circuits that can dynamically respond to cues of drug resistance. For instance, SynNotch-based CAR systems can restrict cytotoxic activity to dual-antigen environments, thereby minimizing off-target toxicity and addressing antigen heterogeneity, a common cause of CAR-T treatment failure in ovarian cancer (36).

Additionally, synthetic promoters responsive to hypoxia, inflammatory cytokines, or estrogen receptor activation are being developed to regulate therapeutic gene expression in drug-resistant microenvironments. These designs enable the combinatorial use of hormone therapies or checkpoint inhibitors, thereby optimizing the spatiotemporal control of therapeutic payloads. Self-amplifying RNA systems and base editors are also under exploration for non-disruptive correction of resistance mutations (BRCA1 reversion, TP53 dysfunction) (37).

### 4.2 Tumor microenvironment challenges

TME presents both physical and molecular barriers to therapy, particularly in cases of chemoresistant ovarian and endometrial tumors. Dense stroma, hypoxia, and high levels of TGF-β contribute to immune exclusion, suppressing the activity of engineered T or NK cells. These factors also drive epithelial-mesenchymal transition (EMT), a known contributor to platinum resistance and metastasis (38).

To overcome these difficulties, strategies such as co-administration of collagenase, nanoparticle-based TGF-β inhibitors, and armored CAR-T cells capable of secreting IL-12 or DNase I are under investigation, which improve drug penetration and enhance immune access to persistent tumor islets. Gene therapies targeting matrix metalloproteinases (MMPs), angiogenesis factors, or immune checkpoints within the TME are also being pursued as adjuncts to standard chemotherapeutic regimens. Such modulation may reinstate chemosensitivity and prolong responses to agents like paclitaxel or bevacizumab (39).

### 4.3 Regulatory and clinical challenges

Despite the therapeutic promise, gene and cell therapies for gynecological applications face complex regulatory landscapes. Safety concerns include insertional mutagenesis, off-target gene edits, and immunogenicity of engineered cells. For reproductive-age women, reproductive toxicity, placental transmission, and germline editing remain under strict scrutiny, especially when delivery is intrauterine or intraperitoneal (40).

Manufacturing and scalability present additional bottlenecks. Autologous CAR-T cell production is time-consuming and resource-intensive, limiting accessibility in low-resource settings where gynecologic cancers are most prevalent. To address this issue, allogeneic “off-the-shelf” CAR and NK cell platforms are being developed in conjunction with point-of-care processing units (41).

**Figure 2:**
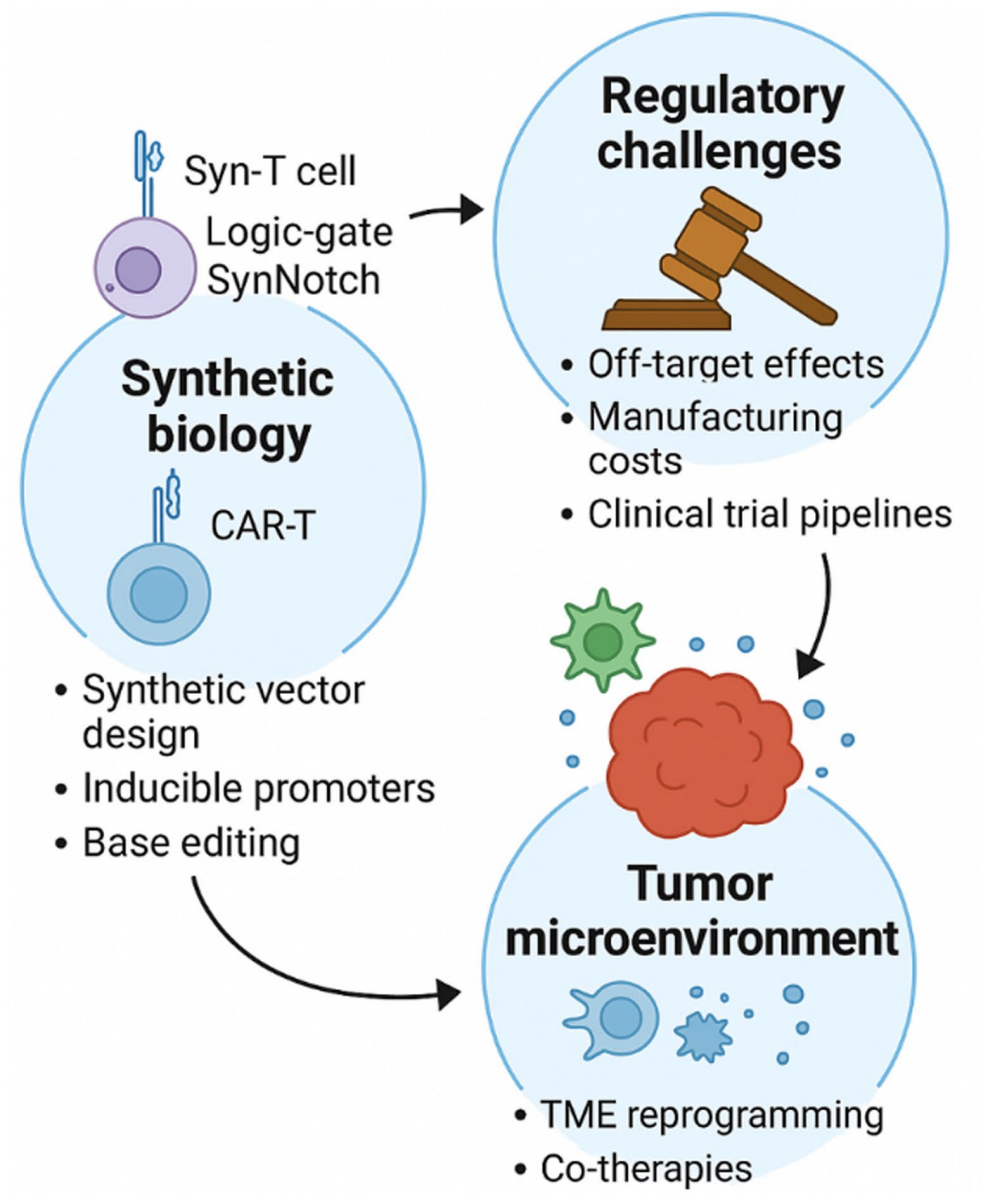
Schematic overview of the translational challenges and their suggested strategies, including synthetic vector design, TME reprogramming, and clinical regulatory strategies driving the clinical advancement of gene- and cell-therapies for gynecologic disorders.

Clinical trial pipelines also lag behind hematologic indications. Many gynecological trials are early-phase or exploratory, lacking large-scale randomized data. Multimodal trial designs, integrating gene or cell therapies with conventional drugs, are necessary to clarify sequencing, synergy, and the reversal of resistance (42). Figure 2 displays an overview of translational challenges and emerging novelties in synthetic biology, TME modulation, and regulatory solutions in the clinical regulatory frameworks supporting the implementation of gene- and cell-based interventions in gynecology. Additionally, a summary of gene- and cell-based therapeutic trials for gynecologic disorders is presented (see Table 2).

**Table 2:** Selected clinical trials of gene- and cell-therapies in gynecological disorders (32).

Therapy type	Target disease	Target gene/antigen	Delivery platform	Trial phase	Resistance context	Status
CRISPR/Cas9	Cervical cancer	HPV16 E6/E7	Lentiviral vector	Preclinical	Restores p53; sensitizes to cisplatin	Ongoing
siRNA + Paclitaxel	Ovarian cancer	BCL2	Lipid nanoparticle	Phase I	Overcomes multidrug resistance	Recruiting
CAR-T (anti-MUC16)	Ovarian cancer	MUC16 (CA125)	mRNA electroporation	Phase I	Targets platinum-resistant clones	Active
CAR-NK (anti-EpCAM)	Endometrial cancer	EpCAM	Retroviral vector	Phase I/II	Immune escape in checkpoint failure	Planned
MSC transplantation	Asherman’s syndrome	n/a	Intrauterine infusion	Phase II	Alternative to repeated hormonal therapy	Completed
TGF-βi gene therapy	Uterine fibroids	TGFBR2	Adenoviral vector	Preclinical	Enhances response to SPRMs	Completed

## 5 Future recommendations and research gaps

As gene and cell therapies transition from the preclinical stage to clinical application in gynecological diseases, several research directions have emerged that could refine their role in managing drug resistance and enhancing therapeutic precision. One of the most transformative avenues is the integration of artificial intelligence (AI) into therapy design. AI-enabled platforms can analyze multi-omics datasets to identify resistance-driving mutations, predict CRISPR off-target effects, and optimize CAR-T or RNAi constructs for patient-specific interventions (43). This predictive power is especially valuable in high-grade serous ovarian cancer, where chemoresistance evolves rapidly due to genomic instability.

Personalization remains a central theme in future therapeutic frameworks. Individual tumor mutational profiles, hormone receptor status, and TME characteristics can inform the selection of guide RNAs, delivery vectors, or cellular constructs for targeted therapies. Similarly, personalized delivery of miRNA or lncRNA through stem cell-derived exosomes is responsive to progesterone or aromatase inhibitors (44).

To enhance therapeutic outcomes, combination strategies represent another key area of research, aiming to integrate cellular therapies with hormonal, molecular, or immune-therapeutic modulators. However, the main questions, including the optimal sequencing of therapies, suitable dosing schedules, and the use of biomarkers to guide patient selection, remain unanswered. To rigorously assess these integrated strategies, clinical trials must be carefully designed, particularly in the context of resistant or recurrent gynecologic tumors, where treatment options are limited.

As advanced therapies are still limited by cost and geography, future efforts should prioritize reducing expenses by streamlining manufacturing processes, developing scalable allogeneic products, and ensuring integration into resource-limited settings where the burden of gynecologic tumors is uppermost.

The application of novel biomaterials (e.g., biodegradable scaffolds and stimuli-responsive hydrogels) may enhance local gene or cell delivery while minimizing adverse cytotoxicity. These novelties could offer a new avenue for localized drug-resistant lesions, post-surgical adhesions, or intrauterine fibrosis (45).

## 6 Conclusion

Gene and cell therapies offer targeted interventions for conditions that frequently evade conventional treatments, and they are poised to transform treatment for gynecological disorders. These approaches handle both the root causes of disease and the pressing issue of drug resistance, from CRISPR-based correction of oncogenic mutations in cervical and ovarian cancers to MSC-driven regeneration in endometrial disorders.

Integrating these novel strategies with recognized hormonal, chemotherapeutic, and immunologic treatments upturns the possibility of achieving synergistic therapeutic effects. Gene therapies can enable the restoration of sensitivity to Pt-based drugs, mitigate inflammatory responses in hormone-resistant diseases, and modulate cell death mechanisms. At the same time, engineered T and NK cells can eliminate tumor clones, while advanced delivery systems and synthetic vectors improve targeting, extend therapeutic efficacy, and minimize toxicity.

Despite promising results, significant challenges remain, including overcoming immune evasion, ensuring scalable manufacturing, refining delivery techniques, and guaranteeing long-term safety and stability. Furthermore, ethical, regulatory, and reproductive concerns require careful consideration, particularly for gene editing interventions targeting reproductive tissues. Through multidisciplinary partnerships, a focus on patient-centered approaches, well-designed clinical trials, and gene and cell therapies, we possess strong capabilities to drive personalized drug development forward in women’s reproductive health.

## References

[1] Li C Zhang L Liu C He X Chen M Chen J. Lipophilic grape seed proanthocyanidin exerts anti-cervical cancer effects in HeLa cells and a HeLa-derived xenograft zebrafish model. Antioxidants (Basel). (2022) 11:422. doi: 10.3390/antiox11020422 35204304 PMC8869705

[2] Zhu P Tatar O Haward B Griffin-Mathieu G Perez S Smith L. Assessing Canadian women's preferences for cervical cancer screening: a brief report. Front Public Health. (2022) 10:962039. doi: 10.3389/fpubh.2022.962039 35968487 PMC9366717

[3] Rodrigues LLS Morgado MG Sahasrabuddhe VV De Paula VS Oliveira NS Chavez-Juan E. Cervico-vaginal self-collection in HIV-infected and uninfected women from Tapajós region, Amazon, Brazil: high acceptability, hrHPV diversity and risk factors. Gynecol Oncol. (2018) 151:102–10. doi: 10.1016/j.ygyno.2018.08.004 30087059 PMC6151287

[4] Kumar K Bose S Chakrabarti S. Identification of cross-pathway connections via protein–protein interactions linked to altered states of metabolic enzymes in cervical cancer. Front Med. (2021) 8:736495. doi: 10.3389/fmed.2021.736495 PMC8591138 34790674

[5] Perrone E Tudisco R Pafundi PC Guido D Ciucci A Martinelli E. What’s beyond BRCA mutational status in high grade serous ovarian cancer? The impact of hormone receptor expression in a large BRCA-profiled ovarian cancer patient series: a retrospective cohort study. Cancers (Basel). (2022) 14:4588. doi: 10.3390/cancers14194588 36230510 PMC9559459

[6] Lai H Guo Y Tian L Linxiang W Li X Yang Z. Protein panel of serum-derived small extracellular vesicles for the screening and diagnosis of epithelial ovarian cancer. Cancers (Basel). (2022) 14:3719. doi: 10.3390/cancers14153719 35954383 PMC9367436

[7] Kim HJ Lee S Oh Y-S Chang HK Kim YS Hong SH. Humanized anti-hepatocyte growth factor monoclonal antibody (YYB-101) inhibits ovarian cancer progression. Front Oncol. (2019) 9:571. doi: 10.3389/fonc.2019.00571 31355133 PMC6631954

[8] Huo X Sun H Qian Q Ma X Peng P Mei Y. CYP27B1 downregulation: a new molecular mechanism regulating EZH2 in ovarian cancer tumorigenicity. Front Cell Dev Biol. (2020) 8:561804. doi: 10.3389/fcell.2020.561804 33163485 PMC7591459

[9] Qian J LeSavage BL Hubka KM Ma C Natarajan S Eggold JT. Cancer-associated mesothelial cells promote ovarian cancer chemoresistance through paracrine osteopontin signaling. J Clin Invest. (2021) 131:e146186. doi: 10.1172/JCI146186 34396988 PMC8363279

[10] Bae S-J Jo Y Cho MK Jin J-S Kim J-Y Shim J. Identification and analysis of novel endometriosis biomarkers via integrative bioinformatics. Front Endocrinol. (2022) 13:942368. doi: 10.3389/fendo.2022.942368, PMID: 36339397 PMC9630743

[11] He Y Hung SW Liang B Zhang R Gao Y Chu CY. Receptor tyrosine kinase inhibitor sunitinib as novel immunotherapy to inhibit myeloid-derived suppressor cells for treatment of endometriosis. Front Immunol. (2021) 12:641206. doi: 10.3389/fimmu.2021.641206, PMID: 34367125 PMC8340010

[12] Moyo MB Parker JB Chakravarti D. Altered chromatin landscape and enhancer engagement underlie transcriptional dysregulation in MED12 mutant uterine leiomyomas. Nat Commun. (2020) 11:1019. doi: 10.1038/s41467-020-14701-6 32094355 PMC7040020

[13] Jiang L Tong D Li Y Liu Q Liu K. Application of single-port laparoscopic surgery in myomectomy. Front Oncol. (2021) 11:722084. doi: 10.3389/fonc.2021.722084, PMID: 34631550 PMC8497760

[14] Zheng Y Zhang H Sun H. Metformin inhibits the proliferation and invasion of ovarian cancer cells by suppressing tripartite motif-containing 37-induced tumor necrosis factor receptor-associated factor 2 ubiquitination. Cancer Sci. (2022) 113:3776–86. doi: 10.1111/cas.15524, PMID: 35950370 PMC9633302

[15] Yin Z Low H-Y Chen BS Huang K-S Zhang Y Wang Y-H. Risk of ankylosing spondylitis in patients with endometriosis: a population-based retrospective cohort study. Front Immunol. (2022) 13:877942. doi: 10.3389/fimmu.2022.877942, PMID: 35784295 PMC9240188

[16] Yao L Wu J Gu W Huang Y Tong Z Huang L. Exosome-liposome hybrid nanoparticles deliver CRISPR/Cas9 system in MSCs. Adv Sci (Weinh). (2018) 5:1700611. doi: 10.1002/advs.201700611 29721412 PMC5908366

[17] Öztürk BE Johnson ME Kleyman M Turunç S He J Jabalameli S. scAAVengr, a transcriptome-based pipeline for quantitative ranking of engineered AAVs with single-cell resolution. eLife. (2021) 10:e64175. doi: 10.7554/eLife.64175 34664552 PMC8612735

[18] Hassan MH Othman EE Hornung D Al-Hendy A. Gene therapy of benign gynecological diseases. Adv Drug Deliv Rev. (2009) 61:822–35. doi: 10.1016/j.addr.2009.04.023, PMID: 19446586 PMC4477532

[19] Garg P Malhotra J Kulkarni P Horne D Salgia R Singhal SS. Emerging therapeutic strategies to overcome drug resistance in cancer cells. Cancers (Basel). (2024) 16:2478. doi: 10.3390/cancers16132478, PMID: 39001539 PMC11240358

[20] Chehelgerdi M Chehelgerdi M Khorramian-Ghahfarokhi M Shafieizadeh M Mahmoudi E Eskandari F. Comprehensive review of CRISPR-based gene editing: mechanisms, challenges, and applications in cancer therapy. Mol Cancer. (2024) 23:9. doi: 10.1186/s12943-023-01925-5 38195537 PMC10775503

[21] Wang D Tai PWL Gao G. Adeno-associated virus vector as a platform for gene therapy delivery. Nat Rev Drug Discov. (2019) 18:358–78. doi: 10.1038/s41573-019-0012-9, PMID: 30710128 PMC6927556

[22] Zhou W-J Yang H-L Shao J Mei J Chang K-K Zhu R. Anti-inflammatory cytokines in endometriosis. Cell Mol Life Sci. (2019) 76:2111–32. doi: 10.1007/s00018-019-03056-x, PMID: 30826860 PMC11105498

[23] Yamano S Inoue K. Non-viral gene delivery systems in the treatment of oral cancers: a promising future. Nanomedicine. (2025) 20:1995–8. doi: 10.1080/17435889.2025.2503693, PMID: 40366745 PMC12330268

[24] Xiao B Ma L Merlin D. Nanoparticle-mediated co-delivery of chemotherapeutic agent and siRNA for combination cancer therapy. Expert Opin Drug Deliv. (2017) 14:65–73. doi: 10.1080/17425247.2016.1205583, PMID: 27337289 PMC5531052

[25] Liu Y Hu X Han C Wang L Zhang X He X. Targeting tumor suppressor genes for cancer therapy. BioEssays. (2015) 37:1277–86. doi: 10.1002/bies.201500093, PMID: 26445307 PMC8638220

[26] Huang H Huang SY Chen TT Chen JC Chiou CL Huang TM. Cisplatin restores p53 function and enhances the radiosensitivity in HPV16 E6 containing SiHa cells. J Cell Biochem. (2004) 91:756–65. doi: 10.1002/jcb.10769, PMID: 14991767

[27] Bezler M Hengstler JG Ullrich A. Inhibition of doxorubicin-induced HER3-PI3K-AKT signalling enhances apoptosis of ovarian cancer cells. Mol Oncol. (2012) 6:516–29. doi: 10.1016/j.molonc.2012.07.001, PMID: 22841590 PMC5528396

[28] Perillo A Ferrandina G Pierelli L Bonanno G Scambia G Mancuso S. Stem cell-based treatments for gynecological solid tumors. Eur Rev Med Pharmacol Sci. (2005) 9:93–102. 15945498

[29] Zhu X Cai H Zhao L Ning L Lang J. CAR-T cell therapy in ovarian cancer: from the bench to the bedside. Oncotarget. (2017) 8:64607–21. doi: 10.18632/oncotarget.19929, PMID: 28969098 PMC5610030

[30] Cutri-French C Nasioudis D George E Tanyi JL. CAR-T cell therapy in ovarian cancer: where are we now? Diagnostics (Basel). (2024) 14:819. doi: 10.3390/diagnostics14080819, PMID: 38667465 PMC11049291

[31] Madrigal M Janicek MF Sevin BU Perras J Estape R Peñalver M. In vitro antigene therapy targeting HPV-16 E6 and E7 in cervical carcinoma. Gynecol Oncol. (1997) 64:18–25. doi: 10.1006/gyno.1996.4515, PMID: 8995542

[32] Cao Y Wang X Jin T Tian Y Dai C Widarma C. Immune checkpoint molecules in natural killer cells as potential targets for cancer immunotherapy. Signal Transduct Target Ther. (2020) 5:250. doi: 10.1038/s41392-020-00348-8, PMID: 33122640 PMC7596531

[33] Marofi F Al-Awad AS Sulaiman Rahman H Markov A Abdelbasset WK Ivanovna Enina Y. CAR-NK cell: a new paradigm in tumor immunotherapy. Front Oncol. (2021) 11:673276. doi: 10.3389/fonc.2021.673276 34178661 PMC8223062

[34] Rungsiwiwut R Virutamasen P Pruksananonda K. Mesenchymal stem cells for restoring endometrial function: an infertility perspective. Reprod Med Biol. (2020) 20:13–9. doi: 10.1002/rmb2.12339, PMID: 33488279 PMC7812475

[35] Hade MD Suire CN Suo Z. Mesenchymal stem cell-derived exosomes: applications in regenerative medicine. Cells. (2021) 10:1959. doi: 10.3390/cells10081959, PMID: 34440728 PMC8393426

[36] Bonfá G Blazquez-Roman J Tarnai R Siciliano V. Precision tools in immuno-oncology: synthetic gene circuits for cancer immunotherapy. Vaccine. (2020) 8:732. doi: 10.3390/vaccines8040732, PMID: 33287392 PMC7761833

[37] Greenshpan Y Sharabi O Ottolenghi A Cahana A Kundu K Yegodayev KM. Synthetic promoters to induce immune-effectors into the tumor microenvironment. Commun Biol. (2021) 4:143. doi: 10.1038/s42003-021-01664-7, PMID: 33514819 PMC7846768

[38] Blanc-Durand F Xian W Tan LC Tan DSP. Targeting the immune microenvironment for ovarian cancer therapy. Front Immunol. (2023) 14:1328651. doi: 10.3389/fimmu.2023.1328651 38164130 PMC10757966

[39] Rafiq S Hackett CS Brentjens RJ. Engineering strategies to overcome the current roadblocks in CAR T cell therapy. Nat Rev Clin Oncol. (2020) 17:147–67. doi: 10.1038/s41571-019-0297-y, PMID: 31848460 PMC7223338

[40] Youssef E Fletcher B Palmer D. Enhancing precision in cancer treatment: the role of gene therapy and immune modulation in oncology. Front Med. (2024) 11:1527600. doi: 10.3389/fmed.2024.1527600, PMID: 39871848 PMC11769984

[41] Anurogo D Luthfiana D Anripa N Fauziah AI Soleha M Rahmah L. The art of bioimmunogenomics (BIGs) 5.0 in CAR-T cell therapy for lymphoma management. Adv Pharm Bull. (2024) 14:314–30. doi: 10.34172/apb.2024.034, PMID: 39206402 PMC11347730

[42] Drakopoulou E Anagnou NP Pappa KI. Gene therapy for malignant and benign gynaecological disorders: a systematic review of an emerging success story. Cancer. (2022) 14:3238. doi: 10.3390/cancers14133238, PMID: 35805007 PMC9265289

[43] Boretti A. The transformative potential of AI-driven CRISPR-Cas9 genome editing to enhance CAR T-cell therapy. Comput Biol Med. (2024) 182:109137. doi: 10.1016/j.compbiomed.2024.109137, PMID: 39260044

[44] Dalmizrak A Dalmizrak O. Mesenchymal stem cell-derived exosomes as new tools for delivery of miRNAs in the treatment of cancer. Front Bioeng Biotechnol. (2022) 10:956563. doi: 10.3389/fbioe.2022.956563, PMID: 36225602 PMC9548561

[45] Hu B Gao J Lu Y Wang Y. Applications of degradable hydrogels in novel approaches to disease treatment and new modes of drug delivery. Pharmaceutics. (2023) 15:2370. doi: 10.3390/pharmaceutics15102370, PMID: 37896132 PMC10610366

